# The risk of stanford type-A aortic dissection with different tear size and location: a numerical study

**DOI:** 10.1186/s12938-016-0258-y

**Published:** 2016-12-28

**Authors:** Yue Shi, Minjia Zhu, Yu Chang, Huanyu Qiao, Yongmin Liu

**Affiliations:** 1 School of Life Science and BioEngineering, Beijing University of Technology, Beijing, 100124 People’s Republic of China; 2 Department of Cardiac Surgery, Beijing Anzhen Hospital, Capital Medical University, Beijing Institute of Heart, Lung and Blood Vessel Diseases, Beijing, 100029 People’s Republic of China

**Keywords:** Stanford type-A aortic dissection, Hemodynamic, Risk assessment

## Abstract

**Background:**

This study is to investigate the influence of hemodynamics on Stanford type-A aortic dissection with different tear size and location, to provide some support for the relationships between the risks (rupture, reverse tearing and further tearing) and tear size and location for clinical treatment.

**Methods:**

Four numerical models of Stanford type-A aortic dissection were established, with different size and location of the tears. The ratio of the area between the entry and re-entry tears(RA) is various within the model; while, the size and the location of the re-entry in the distal descending aorta are fixed. In model A11 and A21, the entry tears are located near the ascending aorta. The RA in these models are 1 and 2, respectively; In the model B11 and B21, the entry tears are located near the proximal descending aorta and the RA in these models are again assigned to 1 and 2, respectively. Then hemodynamics in these models was solved with numerically and the flow patterns and loading distributions were investigated.

**Results:**

The flow velocity of the true lumen in model A21, B21 is lower than that in A11, B11, respectively; the time-averaged wall shear stress (TAWSS) of the false lumen in model A21 and B21 is higher, and for ascending aorta false lumen, A11, A21 are higher than B11, B21, respectively. False lumen intimal wall pressure of A11, A21 are always higher than the true lumen ones.

**Conclusion:**

The variation of the RA can significantly affect the dynamics of blood within the aortic dissection. When the entry tear size is larger than the re-entry tear ones, the false lumen, proximal descending aorta and the wall near re-entry tear are prone to cracking. Entry tear location can significantly alter the hemodynamics of aortic dissection as well. When entry tear location is closer to proximal ascending aorta, false lumen continues to expand and compress the true lumen resulting in the true lumen reduction. For proximal ascending aorta, high pressure in false lumen predicts a higher risk of reverse tear.

## Background

Cardiovascular diseases are the leading causes of death in the world and disease incidence is steadily increasing in recent years [1, 2]. Aortic dissecting aneurysm is one of the most catastrophic cardiovascular emergencies, especially in Stanford type-A aortic dissection which can be acutely complicated by rapid expansion, rupture and further tearing. Theoretically, once Stanford type-A aortic dissection diagnosed, the patient should do emergency surgery immediately, but restricted by geographical, economic and technological conditions, not all patients can receive treatment in time. Moreover, there is no uniform evaluation criterion to judge which specific condition in type-A dissection is more critical.

With the recent development of computer technology, computational fluid dynamics (CFD) has been widely applied to study the dynamics of blood [3–6]. It provides a more effective way to elucidate the mechanism of some vascular diseases (such as aortic dissection) and to predict their progression. It was pointed out from previous studies that hemodynamic parameters such as flow velocity, wall pressure and wall shear stress (WSS) [7–10] have an important correlation with rupture. CFD helps to understand and predict various phenomena in the development of dissecting aneurysm.

There have been many studies on tears of aortic dissection, for example, Rudenick PA Study Group [11] established computer models with different intimal tear sizes, and simulated blood flow inside the dissection and found blood flows more slowly through the bigger tear, but the flow field in the false lumen is more complex; blood flows more quickly through smaller tear as well as simple flow field in the false lumen. Cheng [12] found that flow rate into the false lumen is dependent on both the size and the location of the primary tear. Blood flow into the false lumen increases with increasing tear size and proximal location. Tse [13] thought that relatively high time-averaged wall shear stress (TAWSS, in the range of 4–8 kPa) may be associated with tear initiation and progression. Chen [14] found that, for patients with multiple tears, if stent-grafts occlude all re-entries, inter-luminal blood communication would be effectively reduced and thus induce thrombosis in the false lumen.

The purpose of this article is to reveal the hemodynamics influence of intimal tear size and location on Stanford type-A aortic dissection. We obtained the boundary conditions of the models from clinical data and lumped parameter model of the cardiovascular system. The flow transport in the true and false lumens was solved numerically and the influence of hemodynamics on Stanford type-A aortic dissection of different tear size and location was investigated. We hope to provide some evidence on preoperative planning of type-A aortic dissection for medical personnel.

## Methods

### Acquisition and the numerical model

50 cases of aorta dissection with only one entry and one re-entry tear were selected. The parts of the aortic dissection model with tears were reconstructed based on the computed tomography angiography (CTA) data. The tear areas were established by a measurement tool (Magics17.0, MaterialiseInc., Belgium). The samples were from the ascending aorta thoracic near the aortic arch and the thickness measurement was completed by Electronic Outside Micrometers. The area of tears and the thickness of flap were measured, and the tear locations were computed. Statistics show that the cases with the area ratio of entry and re-entry tears (RA) is 1 nearly accounted for 50%, and the cases with entry tear area larger than the re-entry tear accounted for 30% and the RA of which is close to 2. The average of flap thickness is 1.2 mm.

**Fig. 1:**
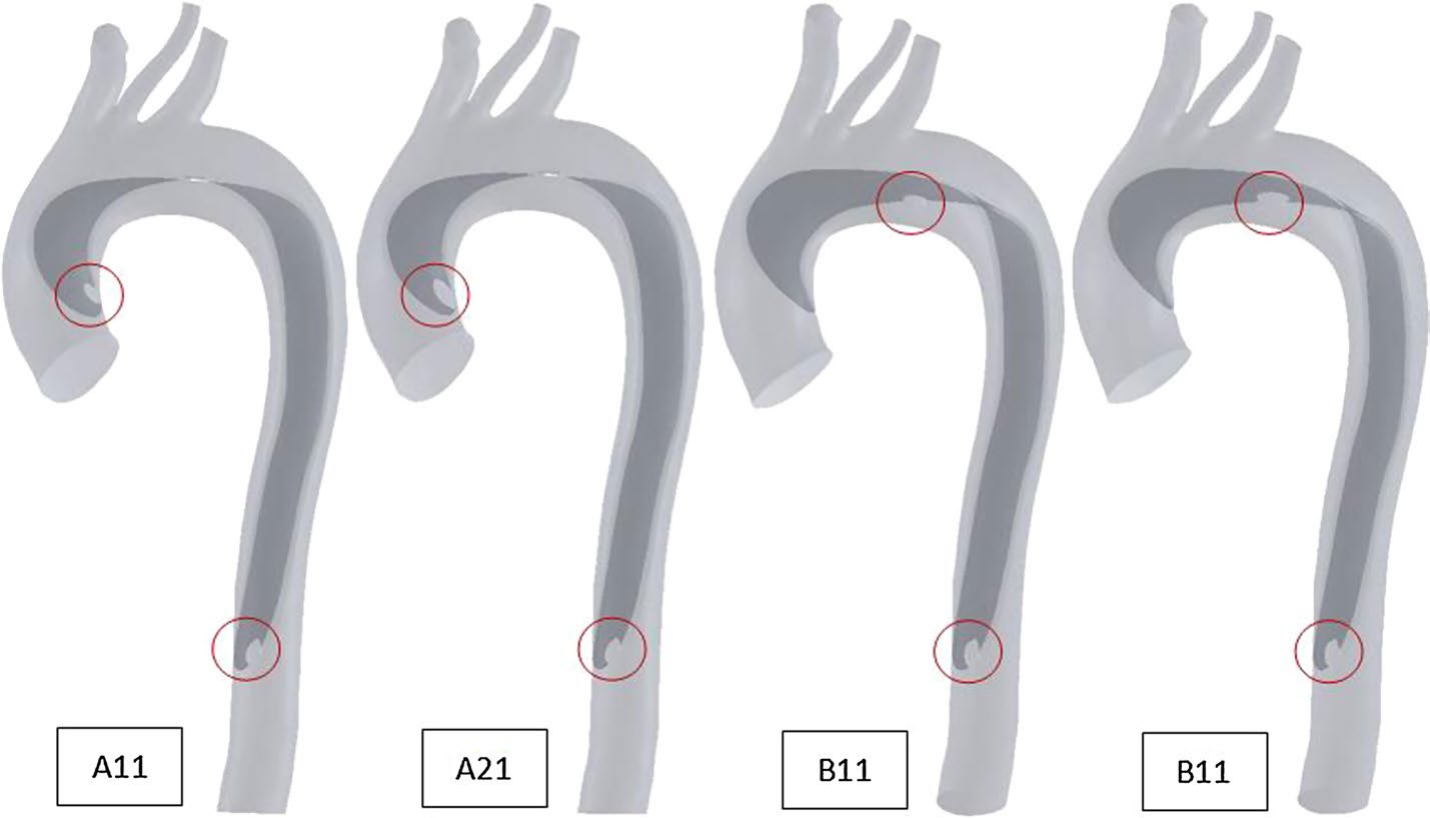
Four Stanford type-A aortic dissection digital models of different size and location of the tear (the size and location of the re-entry in the distal descending aorta are fixed). In model *A11* and *A21*, the entry tears are located near the ascending aorta, and the RA are 1 and 2, respectively; In the model *B11* and *B21*, the entry tears are located near proximal descending aorta and the RA are again assigned to 1 and 2, respectively

The numerical model of the aorta dissection was established based on the CTA data of a normal aorta (in-plane resolution of 512 by 512 pixels with a pixel size of 0.625 mm and slice thickness of 1 mm, total 600 images). Image segmentation and surface reconstruction of the normal aorta were accomplished by a semi-automatic threshold-based segmentation tool (Mimics17.0, MaterialiseInc., Belgium). After smoothed, the format of aorta model was changed into X_T (a kind of parasolid model file format) from STL (stereolithography) by extracting surface function (Geomagic Wrap2015, Geomagic Inc., USA). Starting from the middle of the ascending aorta to the end of the thoracic aorta, along the aorta axis, aorta model was cut off a thin layer with 1.2 mm thickness as the dissection flap. The modification of tear size and location was performed using extruded boss feature by computer aided design (CAD) tool (SolidWorks2015, SolidWorks Inc., France). The final four models are shown in Fig. 1 and geometric model size is shown in Table 1.

**Table 1:** Geometric model size for branches of blood vessels and tear

Variable	Diameter (mm)
Ascending aorta	31.14
Brachiocephalic	11.95
Left common carotid artery	3.64
Left subclavian artery	10.16
Descending aorta	22.32
Re-entry tear (circular)	8.00

In all cases, each branch vascular and re-entry tear are in the same size

### Meshing and elements

A semi-automatic adaptive meshing technique was employed in HyperMeshv10.0 (Altair HyperWorks, Troy, MI, USA) to optimize both computational efficiency and element quality. 4-noded tetrahedral elements were assigned to all models, and element size was set to 0.001 m. The grid was divided into various entrances, exit and intimal regions. The number of elements and node of models meshed as shown in Table 2.

**Table 2:** The numbers of elements and nodes of each model

	A11	A21	B11	B21
Element	1,448,303	1,446,722	1,451,433	1,446,909
Node	252,569	252,209	253,060	252,262

### Boundary conditions and flow models

**Fig. 2:**
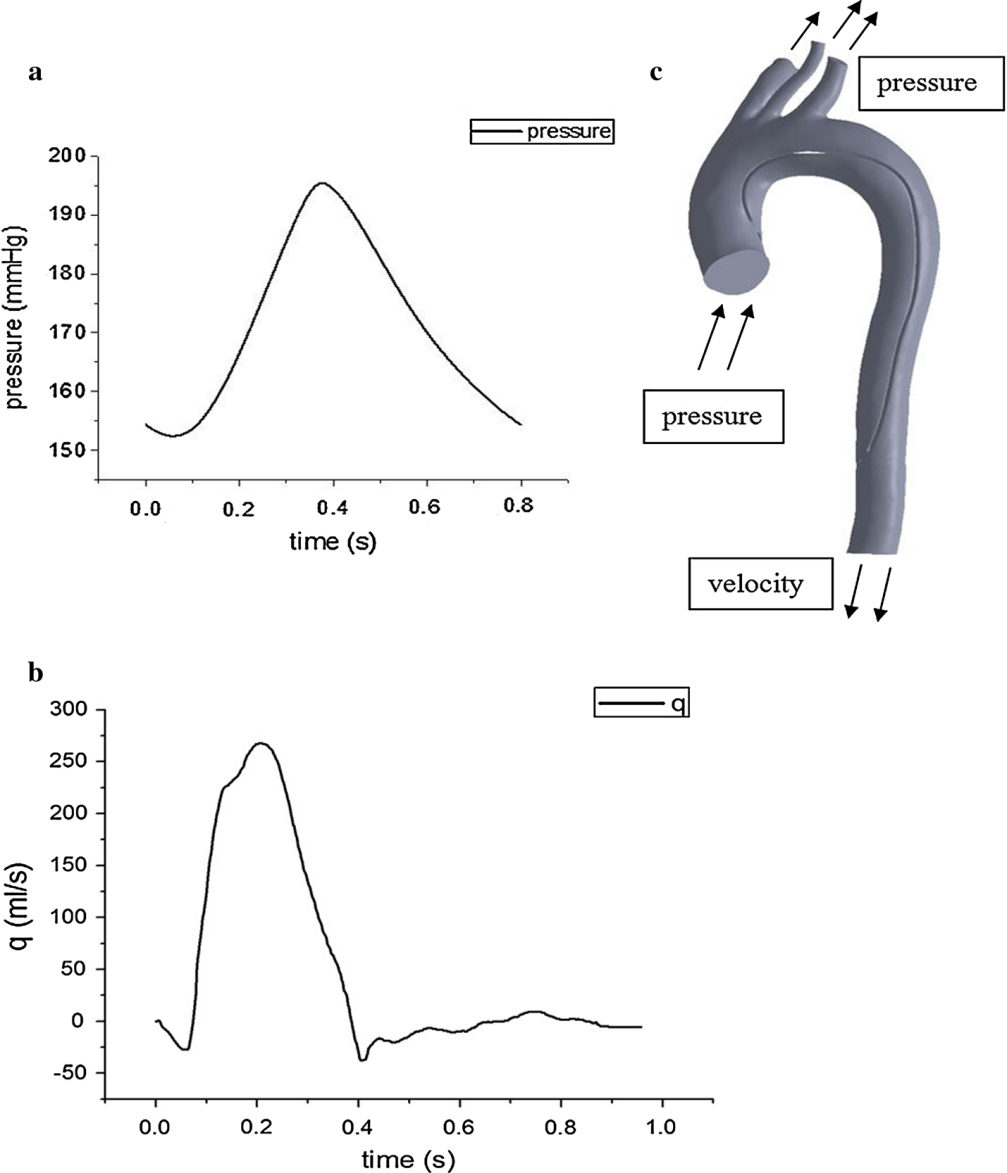
**a** The figure shows the pulsatile waveform of the inlet pressure at the ascending aorta. **b** The figure shows the time-dependent pulsatile waveforms of flow at the descending aorta. **c** The whole aorta model with all boundary conditions shown

The Navier–Stokes equations were solved numerically with a commercial finite-volume-based computational fluid dynamics (CFD) solver (Fluent15.0, ANSYS,Inc., USA). Transient analysis was adopted to investigate the pulsatility of blood flow. The average systolic and diastolic blood pressure of 50 patients was obtained from the clinic. Time-dependent pulsatile waveform of pressure was acquired by fitting the average blood pressure into the curve of normal blood pressure to ensure a consistent trend in blood pressure. The pressure boundary conditions used in this study are consistent with the data in Rapezzi’s work [15]. Time-dependent pulsatile waveform of pressure at the ascending aorta inlet was shown in Fig. 2a. Time-dependent pulsatile waveforms of flow at the descending aorta outlet (Fig. 2b) and brachiocephalic artery outlet, the left subclavian artery outlet, the left common carotid artery outlet, respectively, were obtained from Olufsenet et al.’s [16] work.

Transient analysis was adopted to investigate the pulsatility of blood flow. It was treated that blood is incompressible, and blood has same kinematic viscosity and density of Newtonian fluid [17] with a dynamic viscosity of 3.5 m Pa and a density of 1050 kg/m^3^ [18–20]. Aortic wall was assumed to be rigid and therefore no-slip condition was applied at the aortic wall. In this study, the blood flow in the aortic dissection is unsteady. The maximum Reynolds numbers (Re_max_) in our models is 3208, and the average Reynolds numbers (Re_ave_) based on the average flow velocity (V_ave_) and average hydraulic diameter (D_h, ave_) at peak systole is 944. The Womersley numbers (α) based on the D_h, ave_ is 23.9. So the blood flow is assumed to be laminar [21–23]. The calculation time step and cardiac cycle were set to 0.01 and 0.8 s, respectively. The maximum root mean square residual was given to be 10^−5^, and the maximum number of iterations per time step was set to 200 to ensure adequate accurate results. To minimize the influence of initial flow conditions, all simulations were carried out for four cardiac cycles to achieve a periodic solution, and the results presented here were obtained in the forth cycle.

### RRT

Relative residence time (RRT) refers to the duration that a particle spends to flow through a certain area. If the residence time is short, it means the possibility of particle deposition in the vessel pipe wall is small. The inverse phenomena could be seen when the possibility of particle deposition is large. In the separated, recirculating flow, the RRT is highest [24].

1 $$ {\text{TAWSS}}\;{ = }\;\frac{1}{T}\int_{0}^{T} {WSS\;dt} $$

2 $$ {\text{RRT}}\;{ = }\;\frac{1}{\delta } = 1/\{ (1\,{-}\,2OSI){\text{TAWSS\} }} $$

where oscillatory shear index (OSI) was calculated as Eq. 3 [25]:

3 $$ OSI = \frac{1}{2}\left( {1-\frac{{\left| {\int_{0}^{T} {\tau_{w} dt} } \right|}}{{\int_{0}^{T} {\left| {\tau_{w} } \right|dt} }}} \right) $$

where *τ*_*w*_ is wall shear stress, *T* is one cardiac cycle.

## Results

### Flow velocity, patternsand ratio

**Fig. 3:**
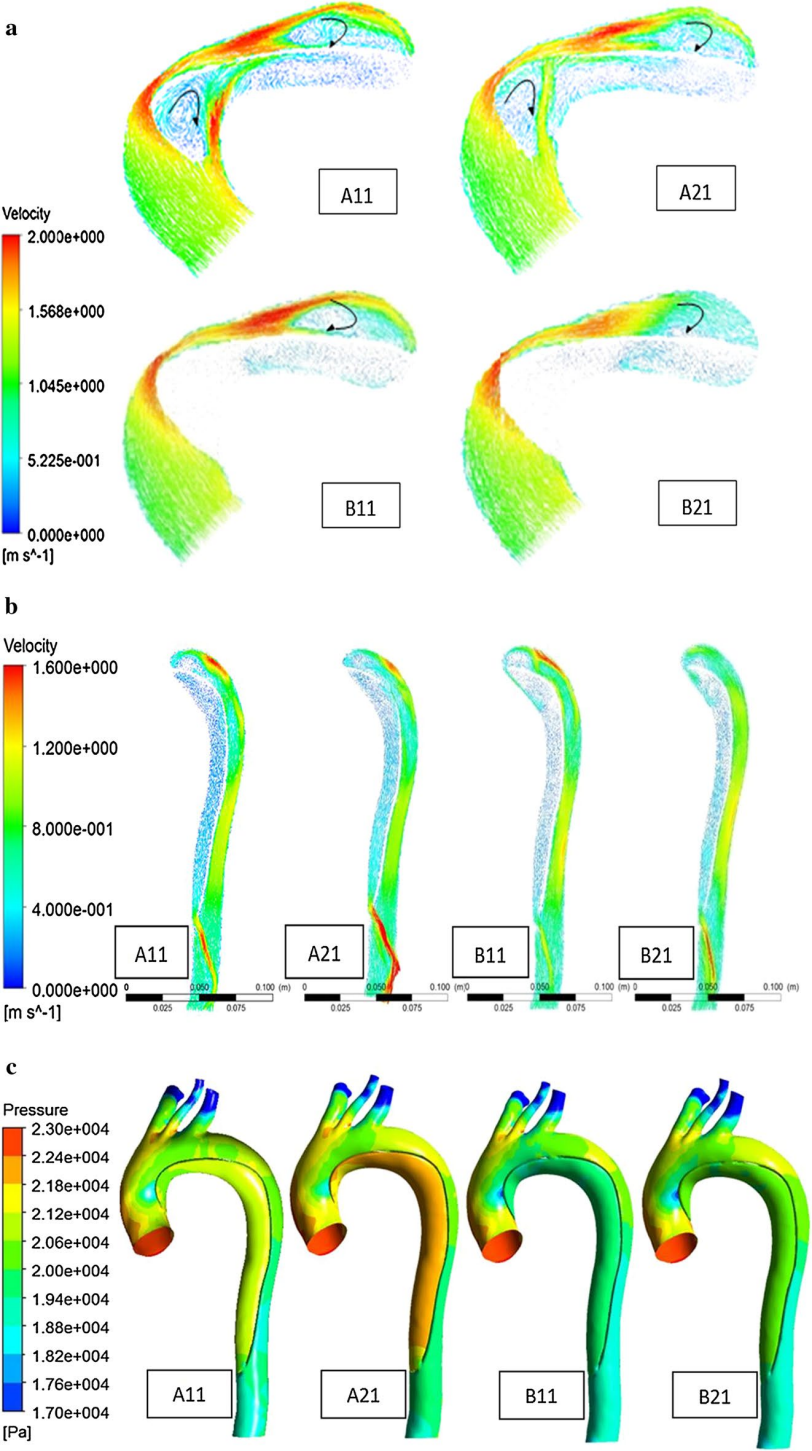
Velocity and wall pressure analysis at systolic peak. **a**, **b** Flow velocity vector map consists of two parts: ascending aorta with aorta arch section (**a**); descending aorta section (**b**). Slices cut tears in half and can display true and false lumen intuitively. Trends in flow velocity distribution among all models are similar; however, flow patterns of ascending aorta with aorta arch section in false lumen are quite different. **c** Pressure distributions. The vessel wall pressure gradually decreases from the proximal to the distal end in all models

Velocity vector plots at peak systole are presented in Fig. 3a, b. In all cases, the true lumen near the aortic arch appears vortex as with the blood flow pattern of the normal aortic arch. For A11 and A21 models, the false lumen blood flow appears vortex, and when the blood flow rate is lower, the phenomenon of vortex is more significant. This phenomenon is consistent with the blood flow pattern observed by the AD patient’s aorta Doppler color ultrasound examination [26]. For B11 and B21, stable blood flow can be seen in the false lumen.

In the whole pulse period, blood flow velocity of the true lumen is greater than in the false lumen. When the blood flows from the aortic arch to the thoracic aorta, the peak value of the velocity is deviated from the medial wall gradually, and the velocity is high near the proximal descending aorta. For all cases, flow velocity around the re-entry tear is high, and velocity increases with increasing entry tear size and proximal location as well. The blood vessel wall impacted more seriously by the blood flow with the higher velocity has more chance to be ruptured.

Table 3 illustrates the percentage of blood flow rate entering the true and false lumens. Results demonstrate significant variability of flow rate into the false lumen among the four models. Regardless of where the entry tear is, the entry tear size is larger, and the false lumen blood flow is more. This phenomenon confirmed to Cheng’s [12] findings.

**Table 3:** Percentage of flow rate into the true and false lumens in different AD cases

Flow ratio, %	A11 (%)	A21 (%)	B11 (%)	B21 (%)
False lumen	29.23	42.98	30.90	38.11
True lumen	70.77	57.02	69.10	61.89

Compared with A11 and A21 models, the flow ratio change of B11 and B21 models caused by the RA is smaller. The possible reason for this phenomenon is the location of entry tear. When the entry tear is near the proximal ascending aorta where blood flow velocity is very high and the blood flows straight to entry tear, therefore the influence of tear size is not so significant.

### Pressure distribution

Pressure profile (Fig. 3c) of the aorta dissection wall is drawn at peak systole.The vessel wall pressure gradually decreases from the proximal to the distal end. False lumen wall pressure of A11, A21 is always higher than that of the true lumen, while the B11 and B21 models is opposite; High wall pressure near proximal descending aorta may indicate that this site may be a dangerous area on the wall of aortic dissection. Continuous impaction on the wall may lead to structural damage and the stiffness and elasticity changes of the vascular wall could damage the vascular wall, so that rupture may occur eventually.

**Fig. 4:**
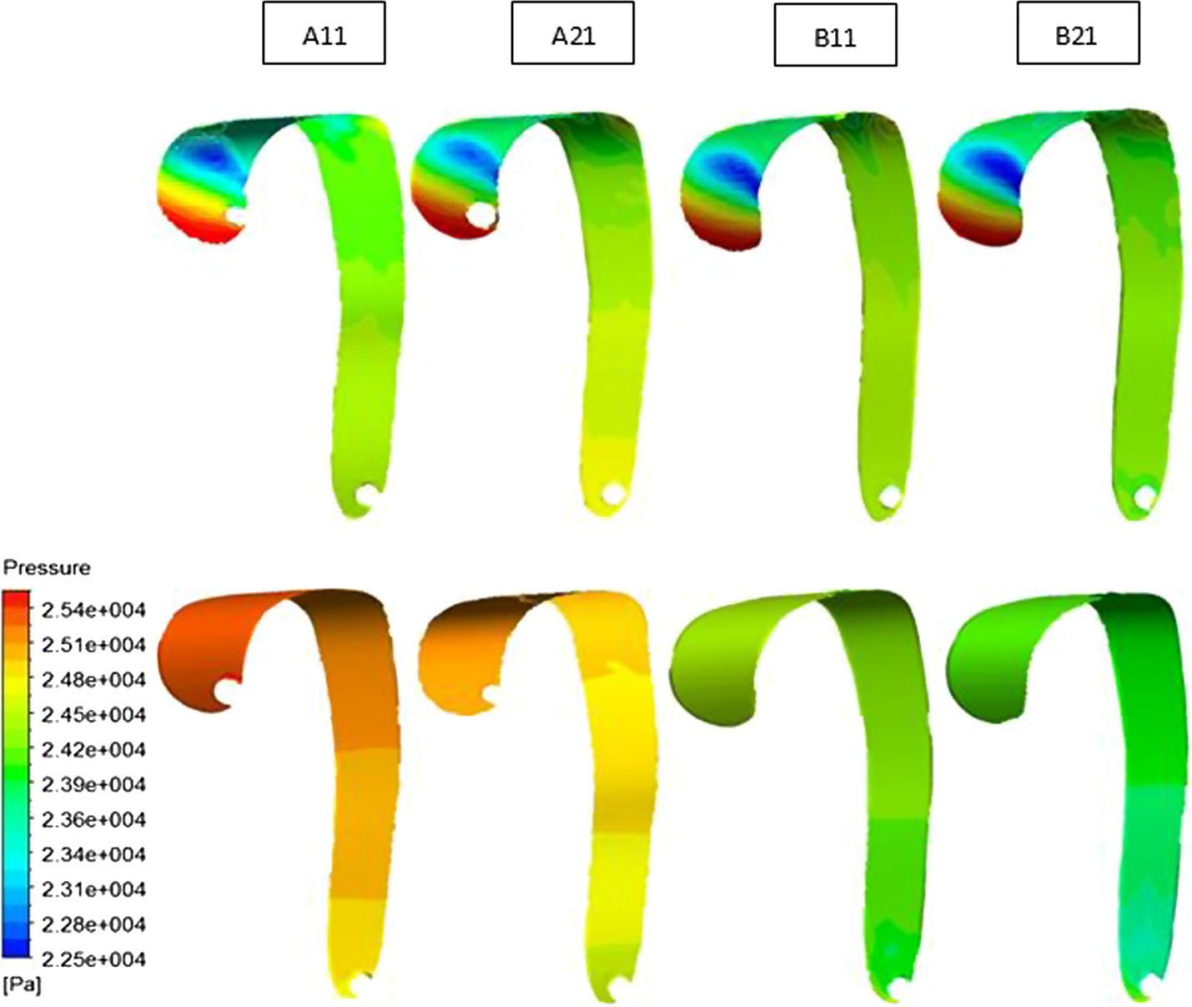
Intima wall pressure contour plots at systolic peak. A view of two sides of intima shows pressure difference in true and false lumen. The *first line* and the *second line* in the figure are the pressure distributions of the true and false lumens sides, respectively

**Fig. 5:**
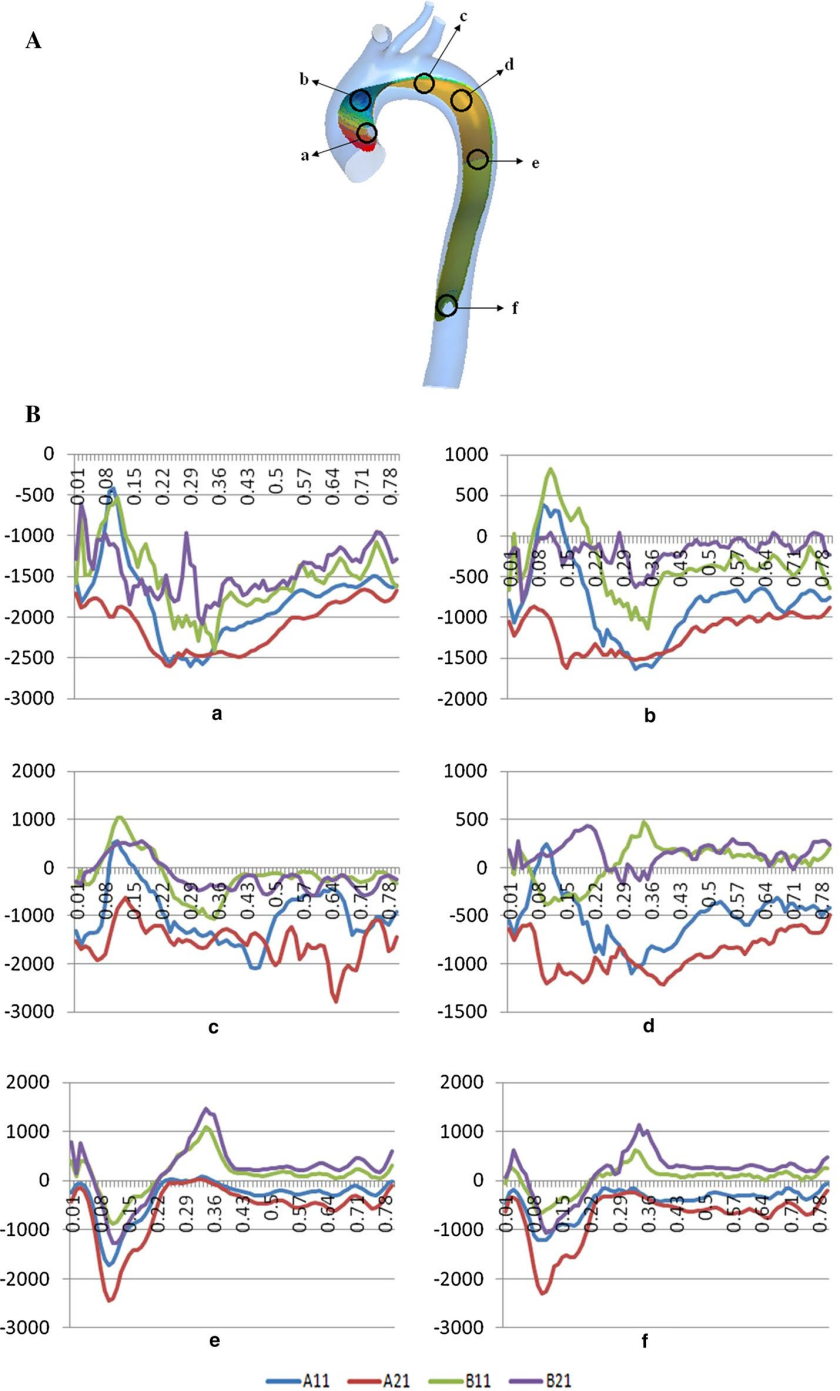
**A** The selection of the key areas on both the true and false lumen sides of intima. The six areas are entry tear area near the proximal ascending aorta *a*, the middle of ascending aorta area *b*, entry tear area near the proximal descending aorta *c*, aorta ligament area *d*, the middle of descending aorta area *e* and re-entry tear area *f*. **B** The true and false lumen intima wall pressure difference of six key small circle areas in the whole cardiac cycle. (Horizontal coordinate: time/s; vertical coordinate: pressure difference/pa)

Pressure contours at systolic peak are presented in Fig. 4, showing two sides of intima (true and false lumens) pressure difference. However, the pressure does not gradually decrease from the proximal to the distal end like the pressure contours of vessel wall. It is found that the wall pressure distribution has a obvious zoning phenomenon, so we select six significant circle areas (the diameter is 16 mm) on the true and false lumen intima wall, as shown in Fig. 5A. We extracted the true and false lumen intima wall pressure difference (the true lumen intima wall pressure minus the false lumen intima wall pressure) of these six areas during the whole cardiac cycle, as shown in Fig. 5B.

During the whole cardiac systolic period, the intima wall pressure difference changes a lot and shows a strong pulsation. Thus, sustained shrinking and stretching of intima will be caused by the periodic fluctuation to adapt the pressure changes at different times of the intima, easily leading to the emergency of the re-entry tears. At the same time, we found that the wall pressure of A11 and A21 models in false lumen almost always is higher than the true lumen ones,resulting that the false lumen has a trend of continual expansion and there is compression on the true lumen in the whole cardiac cycle, especially for A21 model.

### Wall shear stress (WSS)

On account of the wall shear stress cannot be measured directly in clinic, it can be determined by calculating the gradient of velocity field which is got from CFD. However, a better representative of WSS is time averaged wall shear stress (TAWSS), which is obtained by averaging the WSS in a cardiac cycle.

**Fig. 6:**
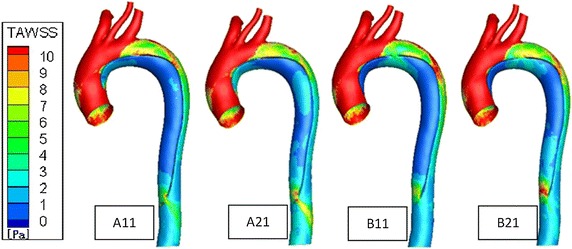
Time-averaged wall shear stress (TAWSS) contour plots in the four models. High TAWSS is found at the entry tear site and the proximal descending aorta of the true lumen

From the TAWSS distribution trends (Fig. 6), the four models are basically the same, TAWSS from the proximal to the distal end of the aorta gradually decreases. The area around the tears partially appears high TAWSS, and TAWSS is higher when entry tear size is larger. Where near the proximal descending aorta on the true lumen wall. TAWSS is high, and A11 and B11 are higher than A21 and B21, respectively. Owning to more blood flows into the true lumen and great changes in the direction of the blood flow, the impact of blood flow on the wall near the proximal descending aorta is larger. The edges of the entry tears in all cases appear localized high TAWSS.

Throughout the whole cardiac circle, TAWSS difference between wall of the true and the false lumen is large. TAWSS of the false lumen is lower (<3. 0 Pa basically) while TAWSS of the true lumen has a greater value and variation range.

### Relative residence time (RRT)

**Fig. 7:**
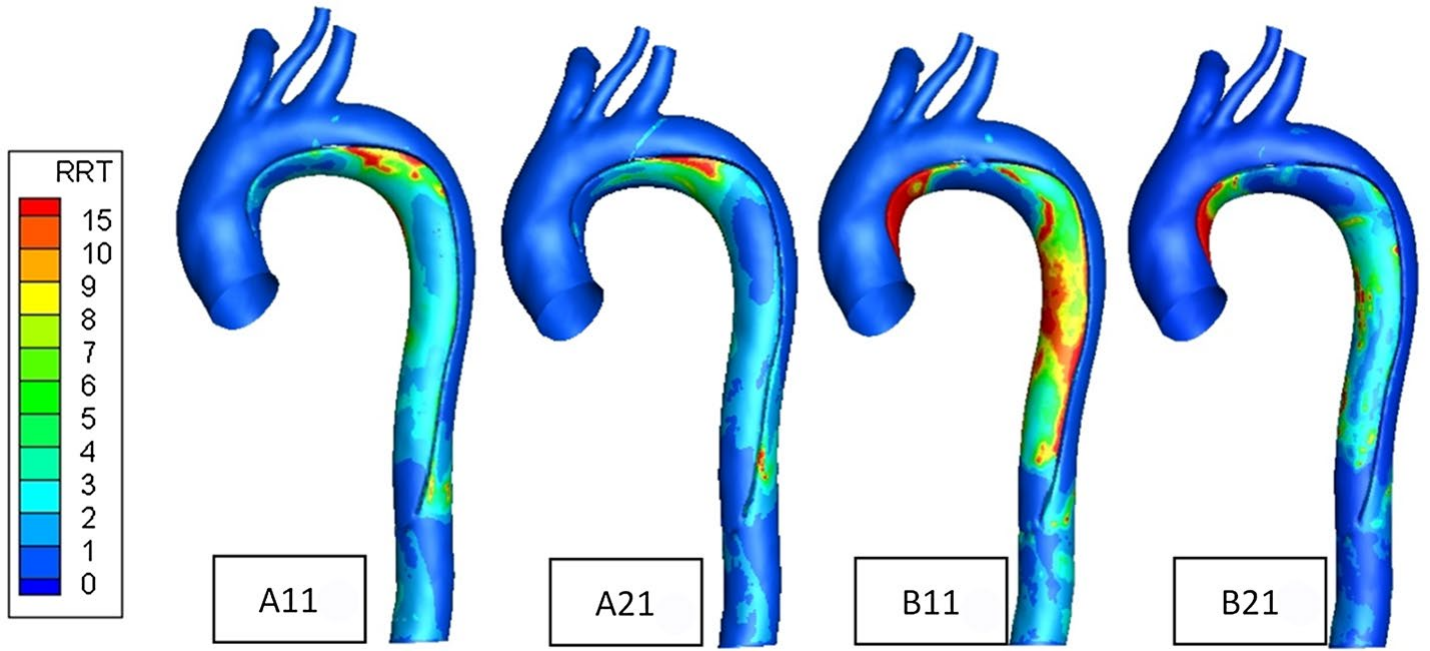
Relative residence time (RRT) contours plots of the four models. High RRT predicts larger possibility of false lumen thrombosis. RRT distribution trends and clinical phenomenon correlate well with areas of thrombus formation in the false lumen

From the RRT distribution trends (Fig. 7), we found that for A11, A21 models, the area of high RRT is small and only distributesin the false lumen part of aortic proximal descending aorta. For B11, B21 models, high RRT regions mainly distribute on the false lumen, especially the ascending aorta part. Due to almost stagnant blood in the false lumen part of the ascending aorta when the entry tear near the proximal descending aorta, so it may be prone to thrombosis.

## Discussion

This study emphasizes on the hemodynamics in Stanford type-A aortic dissection with different tear size and location, hemodynamic factors such as flow patterns, pressure and WSS, which are difficult to measure in vivo, can be determined through CFD simulations.

The phenomenon of blood flow separation is that the blood flow in the lumen of the same artery (actually the true lumen and false lumen blood flow) is separated by the internal flow, as shown in Fig. 3. This phenomenon could be an indirect sign in the diagnosis of aortic dissection [27]. When the entry tear is close to the proximal location, the false lumen in the ascending aorta appears more turbulent. Also the unstable flow may lead to the intense impaction on vascular wall, and therefore, retrograde ascending aortic dissection may occur.

The analysis on pressure distribution prompts us that vessel wall pressure distribution and incidence of aortic dissection may have some correlations. The results are consistent with that Prokop et al. [28] proposed aortic dissection intimal tear usually occurs on the wall where blood pressure is the highest. As well as that in 1830 Elliotson presented aortic dissection is the most common site of intimal tear occurrence. The inner membrane rupture is horizontal while the outer membrane rupture is longitudinal.

Since a higher false lumen pressure plays a critical role in dilating the false lumen and generating an aortic aneurysm [29], the possible development of aneurysm may occur distally to the entry tear. For ascending aorta, high pressure in the false could have contributed to the retrograde ascending aortic dissection. Compared to A11 and A21 models, the false lumen wall pressure of B11 and B21 models is lower. This may be an inducement for the development of Stanford type-B aortic dissection into Stanford type-A aortic dissection.

In the arterial system, the wall shear stress fluctuation is strong where the low shear stress, reflux and vortex occur. High wall shear stress (>10 Pa) was observed for all assessments in the location of the aortic proximal descending aorta. Endothelial cells are very sensitive to WSS, and the endothelial cells are susceptible to fatigue damage under strong shear stress [30, 31]. Local intimal tear occurs when a strong impact of blood flow. It is lead to gradual stripping intimal expansion in the artery, forming the true and false lumen. So flap only has a part of the media layers and the intimal layers. The WSS sensitivity should be different and maybe more sensitive than normal arterial wall. To prove this point, further work need to be done after the discussion.

Thrombosis is typical phenomenon for false lumen, seen in 50% of cases. If the intimal tear occurs in the presence of thrombus in the aneurysm, blood clots in acute aortic dissection true lumen could also be seen. If the false lumen is re-connected with the true lumen by re-entry tear, which would reduce the pressure in the false lumen to some extent. Then the false lumen increases blood flow leading to reduction of incidence rate of thrombosis [13, 14]. However, considering lower speed of blood in the false lumen and narrowed true lumen, together with no additional endothelial cells on the wall of the false lumen, the chance of thrombosis is relatively large in the false lumen. A larger prospective study may allow us to more accurately define which tear location is associated with spontaneous false lumen thrombosis.

There are limitations in this study that need to be improved by further researches. First: the study ignored the vessel wall deformation and the hemodynamic changes. In consideration of a better physiological approach, the simulation should be done with fluid–structure interaction. Second: only two different entry tear changes in the RA and location were involved in our research. To get more comprehensive and accurate conclusion, we should select more subjects, change the tear numbers and the RA.

## Conclusions

This study focuses on hemodynamic effects of different tear size and location of Stanford type-A aortic dissection. By the finite volume method, we use CFD(computational fluid dynamics) to do hemodynamic numerical simulation, then we accessed to aortic true and false lumens blood flow characteristics and hemodynamics changes of dissection development.

In this study, the following conclusions are obtained. First, the RA variation can significantly affect the hemodynamics of aortic dissection. Numerical simulation results show that the flow rate (velocity) in the true lumen is lower when the entry tear size is larger, and inversely, the flow rate (velocity)in the false lumen is higher. The false lumen,the proximal descending aorta and the wall near re-entry tear are prone to rupture more easily when the entry tear size is larger.

Second, entry tear location can significantly alter the hemodynamic characteristics of aortic dissection as well. When the entry tear is close to the proximal location, false lumen wall pressure is higher than the true lumen. Resulting that the false lumen have a continual expansion trend and there is compression on the true lumen. Wall pressure of the descending aorta in the true and the false lumen is unstable when the entry tear is near to the proximal descending aortic. Therefore, it may cause intimal fracture and further tearing. The ascending aortic false lumen TAWSS value is higher when the entry tear is close to proximal location, indicating that the risk of retrograde ascending aortic dissection needs attention.

## Abbreviations

RA: the ratio of the area between the entry and re-entry tears
TAWSS: the time-averaged wall shear stress
CFD: computational fluid dynamics
CTA: computed tomography angiography
STL: stereolithography
CAD: computer aided design
RRT: relative residence time
